# Supplementary material for: Transcriptome Sequencing Reveals the Mechanism behind Chemically Induced Oral Mucositis in a 3D Cell Culture Model
Source: Int J Mol Sci. 2023 Mar 6;24(5):5058. doi: 10.3390/ijms24055058 (PMC10003620; doi:10.3390/ijms24055058)
Supplement: Supplementary file 1 [file ijms-24-05058-s001.zip › ijms-2217119-supplementary.pdf]

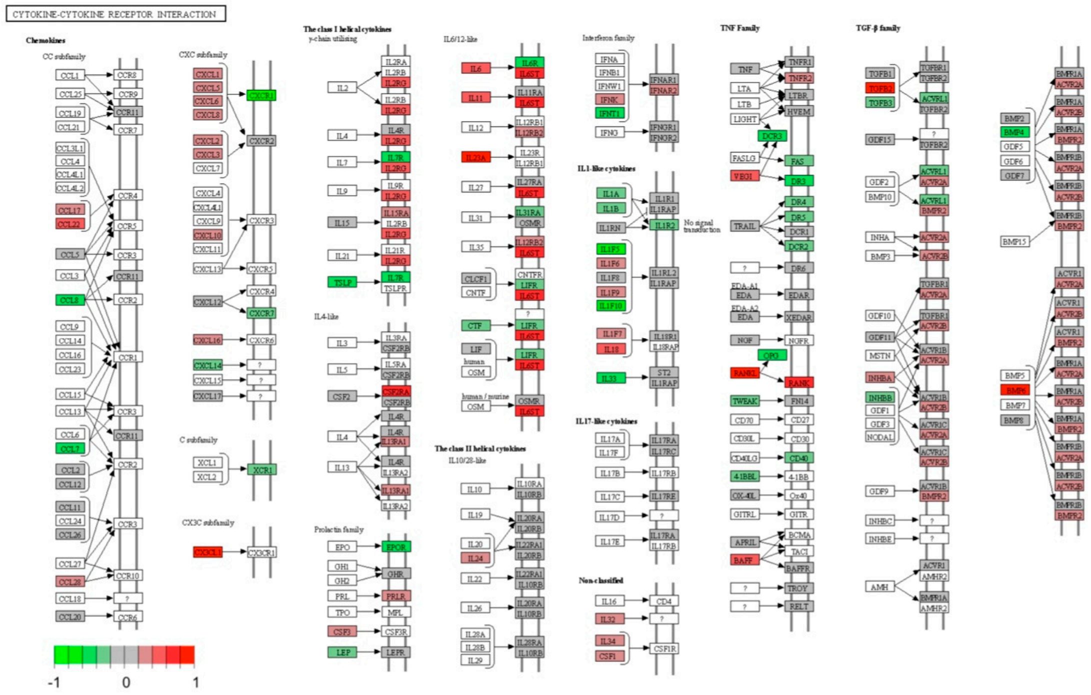

Supplementary Figure S1

Pathview image showing the change in inflammation and cytokines in tissue treated with 64 ng everolimus compared with untreated tissue after 60 h. Green means downregulated and red means upregulated.

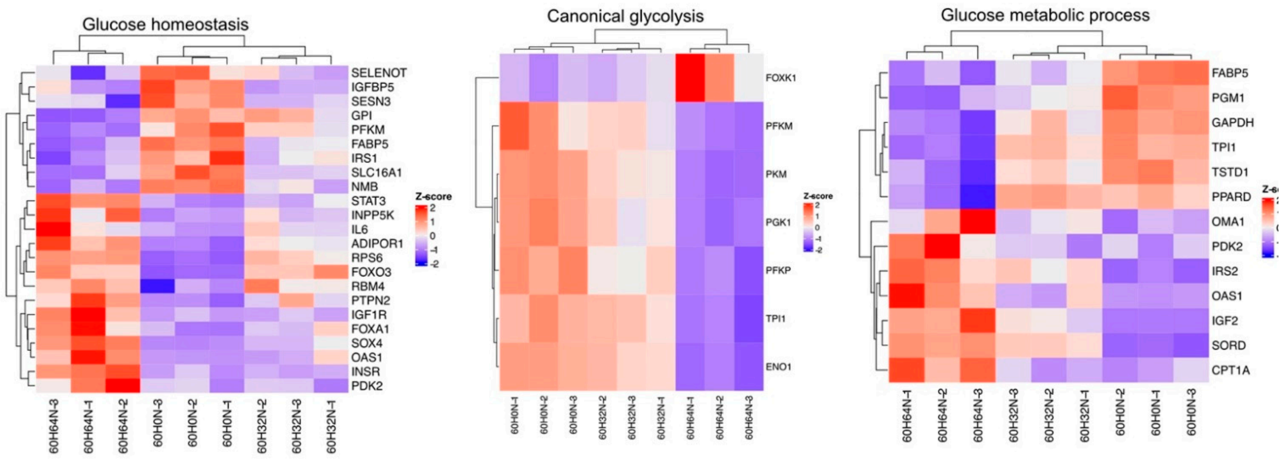

a b c

Supplementary Figure S2

Heatmap illustrating RNA-Seq differential expression for genes associated with glucose homeostasis, canonical glycolysis, and glycose metabolic process.
